# The Homeland in War-Time

**Published:** 1917-11-24

**Authors:** 


### The Hospital

The Workers' Newspaper of Administrative Medicine and Institutional Life, Administration, National Insurance and Health.

No. 1642, Vol. LXIII. SATURDAY, NOVEMBER 24, 1917. PRICE ONE PENNY

### THE HOMELAND IN WAR-TIME.

During the current week the principal newspapers have been publishing a series of articles written by special women correspondents who have been visiting the Front. They will give the people in the Homeland and throughout the Empire an idea of the splendid work which is being done there by women workers of every grade; and there are many grades represented in France. All these workers, from the trained nurses to those who are doing, cheerfully and well, much of the rougher work essential to the full and adequate discharge of the responsibilities devolving upon the military authorities, have brought with them a purposeful energy and splendid courage. These indicate the underlying spirit of the nation at home, seeing that all ranks and positions of life are now represented in the thousands of women workers in question. Our letter-box brings us striking evidence that the same spirit exists amongst the laity of all classes of the population, to an extent which is as remarkable as it is encouraging to those of us, who look with confidence to a new life in the Homeland after the war. This change will unite all classes, and weld them together with loving bonds resting upon an honest desire to secure that, everybody, from the humblest to the highest, shall be enabled to live, work, and maintain themselves, under conditions which secure to them surroundings that, must raise the health of the nation to the highest point, and make it a joy for everybody to live in Great Britain, when they realise the security and freedom, under ideal conditions, which all are privileged to share.

Our home life in England in war-time of late, portions of which are strikingly illustrated this week in the section entitled "Round the Hospitals," affords evidence of how the thoughts of every one of us are continuously with the men, who are fighting our battles in many parts of the world, with such splendid endurance, courage, and resource. How few families are there which have not realised the terrible cost at which we have given of our best in the cause of freedom, the rights of the weaker nations, and the enforcement of the principles which Christ died to uphold. It is but natural that thoughtful people everywhere should lend a hand to help, directly or indirectly, in ministering to the sick and wounded, to the uplifting and after-care of those of our fellows who have been seriously maimed and injured. For them it is our privilege to provide that they shall have all that science and skill can accomplish to help every one of them, whatever his condition, to continue to live a useful, self-supporting life amongst his friends and neighbours, who will proudly and continuously recognise his heroism and prowess. The account we give of the way in which Their Majesties the King and Queen and H.R.H. Princess Mary, with the members of the household, whose names we purposely publish, spent Saturday afternoon the 17th instant, will go straight to the hearts of the British people. The happenings, typical as they were of much that Their Majesties have done during the war, in close association with their people, have an epic character, and are likely to go down in history as a splendid example of the unity, under a limited monarchy, which in this country knits together, in loving sympathy, the Royal Family and all classes of the people at home, and throughout the Empire.

Personal service, in a form which originated under King Edward VII. of blessed memory, has proved in practice an uplifting and uniting force. Its strength will, if possible, be deepened after the war, and it is much that our King and Queen should find in its habitual practice the joyous consolation which has given new life to so many of the original workers for the League of Mercy. The Archbishop of Canterbury many years ago sent his blessing to the organiser of this League, and having regard to the soul-hunger which so many British people are experiencing at the present time, it is an extraordinary circumstance that, with some notable exceptions, the Churches are to-day regarded by many as the great spiritual deserts in our daily life. The laity are ready and hungry for spiritual leadership, and for continuous opportunities actively to expand their spiritual nature. Is the Royal example, as that of a convinced belief in personal service and its teaching, to reach the hearts of all loyal subjects except those whose privilege and supreme duty it is to lead in spiritual matters? The answer must lie with the leaders of the Churches of all denominations. At present a majority of the people feel that the Churches have lost touch with the people and must have fallen on sleep.

